# Human microbiota-associated animal models: a review

**DOI:** 10.3389/fcimb.2025.1644187

**Published:** 2025-08-27

**Authors:** Xiangning Huang, Yunfeng Yu, Na Tian, Jiawang Huang, Xiaoqin Zhang, Rong Yu

**Affiliations:** ^1^ School of Traditional Chinese Medicine, Hunan University of Chinese Medicine, Changsha, China; ^2^ Department of Endocrine, The First Hospital of Hunan University of Chinese Medicine, Changsha, China; ^3^ Department of Rehabilitation, Hunan Provincial People’s Hospital, Changsha, China

**Keywords:** human microbiota-associated animal models, fecal microbiota transplantation, gut microbe-host interactions, microbiome, engraftment, procedure

## Abstract

Human microbiota-associated (HMA) animal models have become indispensable tools for investigating microbe-host interactions and disease pathogenesis. However, standardization challenges persist across different research groups when such models are used in fecal microbiota transplantation (FMT) protocols. Establishing a successful HMA model involves multiple stages, including donor screening, fecal suspension preparation, recipient preparation, and FMT. The outcomes of these stages are influenced by donor characteristics, recipient type, microbial viability, and dietary factors. This review examined the critical components of HMA model production, including the inclusion and exclusion criteria for human donors, collection time and processing methodology for fecal samples, recipient animal preparation strategies, and FMT regimens with engraftment validation. The key findings revealed that short-term antibiotic, probiotic, or laxative use constitutes an essential donor exclusion criterion. The time and method of fecal collection should be standardized as much as possible. Fecal samples should be processed as soon as possible, in anaerobic environments, with the addition of suitable protectants if they must be preserved at low temperatures. Microbial community profiling via 16S rRNA gene sequencing represents the primary method for analyzing microbiome composition and verifying microbiota engraftment efficacy throughout FMT procedures. The most commonly used recipients for HMA modeling included germ-free and pseudo-germ-free animals generated through antibiotic-mediated microbiota depletion. Although FMT with a single gavage of fecal suspension proved sufficient for model establishment, multiple frequencies and longer FMT durations significantly improved the efficiency of donor microbiota colonization. Overall, these findings are expected to aid the establishment of a standardized and reproducible protocol for preparing HMA models.

## 1 Introduction

The microbiota constitutes a complex ecosystem of microorganisms that encompasses bacterial, archaeal, eukaryotic, and viral taxa, each occupying specific ecological niches (Marchesi and Ravel, 2015). These microorganisms demonstrate a ubiquitous natural distribution, with humans serving as one of their primary hosts. Long-term coevolution has cultivated mutualism between humans and their microbiota—particularly within the gastrointestinal tract, where ~95% of endogenous microbes reside. A 2010 metagenomic sequencing analysis revealed that the total human gut microbiome genome exceeds its genomic content by ~150× (Qin et al., 2010). As of 2019, researchers have identified nearly 2,000 novel microbial species in the human intestine (Almeida et al., 2019). Subsequent studies have estimated that the ratio of bacterial to human cells in the adult human body is approximately 1.3:1 (Sender et al., 2016). Recent advancements in multi-omics assay profiling have elucidated the important impact of the microbiome on host health and disease (Integrative HMP (iHMP) Research Network Consortium, 2014). The gut microbial consortium mediates essential physiological functions such as immunological homeostasis, colonization resistance against pathogens, energy metabolism, endocrine regulation, and even certain neurological functions (Lynch and Pedersen, 2016). Dysregulation of the microbial community and abnormalities involving its metabolites have been closely associated with a variety of chronic diseases, including inflammatory bowel disease (Mousa and Al Ali, 2024), certain neuromuscular pathologies (e.g., Alzheimer’s disease (Yang et al., 2024), certain muscular dystrophies (Russo et al., 2024)), metabolic syndromes (e.g., obesity and type 2 diabetes) (Aron-Wisnewsky et al., 2021), and dermatosis (e.g., acne and atopic dermatitis) (Borrego-Ruiz and Borrego, 2024).

The investigation of gut microbe-host interactions offers dual scientific value: elucidating disease mechanisms and pioneering novel diagnostic-therapeutic paradigms. Human microbiota-associated (HMA) animal models have emerged as crucial tools for elucidating the mechanisms underlying microbe-host interactions (Hirayama, 1999; Imaoka et al., 2004; Kibe et al., 2005). Through the transplantation of human microbial communities into recipient animals, HMA models facilitate the longitudinal observation of microbial dynamics or examination of the efficacy of specific therapeutic targets involved in certain interventions (Ridaura et al., 2013). Evidence has demonstrated that HMA models can effectively reconstruct donor microbial signatures and metabolomic profiles (Marcobal et al., 2013). Current applications span four key research domains: the composition of gut microbial consortia, the regulation of gut microbiota in host development, the causal associations between microbes and diseases, and the evaluation of targeted microbiota therapeutic strategies (Sharon et al., 2019). These findings solidify the functional centrality of intestinal microbiomes in terms of maintaining good health. They also provide a scientific basis for microbial interventions that target health benefits across human, animal, and ecological domains.

**Figure 1:**
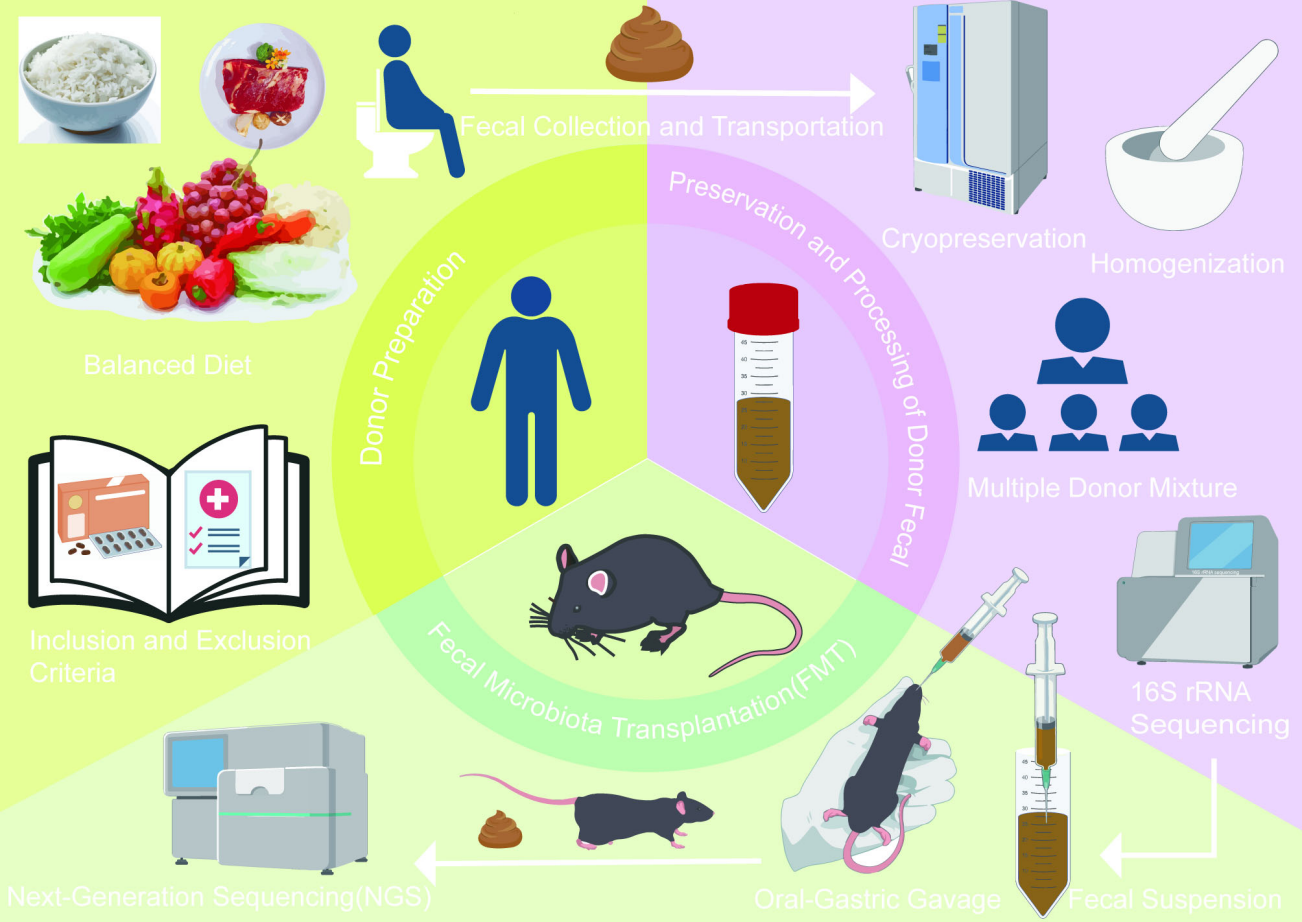
The General procedures of human microbiota-associated (HMA) mice models. Using mice as an example, the general procedures of HMA models primarily involves three steps. Donor Preparation: select human donors with balanced diet who meet predefined inclusion and exclusion criteria. Preservation and Processing of Donor Fecal: collect and transport fecal samples and store them under low-temperature conditions. Standardized fecal suspensions are prepared by diluting, homogenizing, filtering, and pooling fecal samples from multiple donors. Fecal Microbiota Transplantation (FMT): recipient mice are adult germ-free animals or antibiotic-induced pseudo-germ-free models. Following FMT, next-generation sequencing (NGS) is utilized as an effective method to quantify microbial engraftment efficiency.

Despite their scientific utility, HMA animal models derived through fecal microbiota transplantation (FMT) face persistent methodological controversies. The engraftment efficiency of human-derived microbial communities in animal recipients is influenced by several factors. These include the host’s genetic background, gastrointestinal architecture, and behavioral differences—all of which impose certain constraints on HMA animal models (Arrieta et al., 2016). Evidence has indicated that these models risk overestimating the causal associations between microbiomes and disease phenotypes (Walter et al., 2020). Nevertheless, HMA models remain the best choice for investigating host-microbe crosstalk. It remains unclear precisely which methodological refinements in HMA model generation via FMT are required to establish standardized workflows that improve reproducibility and scientific validity. This review highlights key considerations in donor screening, recipient preparation, transplantation protocols, and microbiota validation to enhance HMA model development, experimental reproducibility, and standardization (**Figure 1**).

## 2 Donor preparation

### 2.1 Inclusion and exclusion criteria for human donors

The 2017 European Consensus Conference established donor inclusion and exclusion criteria for clinical fecal microbiota transplantation (FMT), specifying evaluation parameters that included comprehensive medical histories, same-day donation, clinical signs and symptoms, dietary profiling, and laboratory tests (Cammarota et al., 2017). However, standardized protocols for selecting human fecal donors in animal experiments remain undefined, with significant differences remaining in terms of inclusion and exclusion criteria across studies. Current FMT-based human microbiota-associated (HMA) models predominantly use two donor cohorts: healthy individuals, and patients with the diseases being investigated by the study. The inclusion criteria for healthy individuals reported in existing studies mainly included the following aspects: (1) a minimum of 2–12 months without antibiotic exposure (Cherbuy et al., 2019; Kim et al., 2024; Le Bihan et al., 2015; Aluthge et al., 2020; Chung et al., 2012); (2) the elimination of laxative agents for ≥3 months (Brandi et al., 2024; Gérard et al., 2004; Respondek et al., 2013); (3) a omnivorous diet that includes both vegetarian and meat component (Cherbuy et al., 2019; Dong et al., 2021); and (4) the absence of gastrointestinal disorders (Reygner et al., 2020; Tamura et al., 2019; Gobert et al., 2016; Saint-Cyr et al., 2013), recent pathogen (bacterial or parasitic) infection (Lauko et al., 2023; Nagao-Kitamoto et al., 2020, 2016), and acute or chronic illnesses that can alter gut microbe composition (Salandre et al., 2023; Zabolotneva et al., 2023). The most common exclusion criteria included the following: (1) recent (within 1–2 months) exposure to antimicrobials, prebiotics, or probiotics (Zhang et al., 2023; Chen et al., 2020; Xia et al., 2019); (2) active neuropsychiatric disorders including major depression (Gobert et al., 2016; Kaiser et al., 2021; Zabolotneva et al., 2023; Zhan et al., 2024; Zhang et al., 2020); (3) excessive alcoholism or smoking habits (Zabolotneva et al., 2023; Grabrucker et al., 2023; Gobert et al., 2016); and (4) pregnant or lactating (Zabolotneva et al., 2023; Demir et al., 2022; Zhang et al., 2020; Gobert et al., 2016). The inclusion criteria for disease donors typically add the following requirements: clinical manifestations, laboratory tests, and pathological findings that collectively satisfy the diagnostic criteria for the disease (Zhang et al., 2023; Hsu et al., 2023; Fan et al., 2023; Demir et al., 2022; Zhang et al., 2020; Chen et al., 2020; Duan et al., 2019). Exclusion criteria often include: (1) incomplete information (Zhong et al., 2024); (2) the use of medications that could interfere with the experiment (Hutchison et al., 2024; Zhan et al., 2024; Zhong et al., 2024); and (3) comorbidities of chronic or infectious diseases that could affect the study (Hsu et al., 2023; Fan et al., 2023; Chen et al., 2020; Duan et al., 2019; Xia et al., 2019).

Antibiotic exposure and dietary patterns critically influence gut microbiota composition (Dudek-Wicher et al., 2018). Clinical trials have demonstrated that antibiotic administration reduces microbial diversity. It typically takes ≥1.5 months for the intestinal flora of healthy adults to return to near-baseline levels—with a few common taxa remaining undetectable even after 6 months (Palleja et al., 2018). Diet serves as the substrate for the energy used by microbes, with different microbial species differing in their ability to utilize different foods, resulting in different microbial compositions (Flint et al., 2015). Pharmacological interventions such as laxatives induce clearance of intestinal contents, directly altering the microbial community structure (Drago et al., 2019). Probiotic and prebiotic interventions selectively modulate enteric microbial populations, affecting their health-promoting effects (Sanders et al., 2019). Although evidence regarding the impact of alcohol and tobacco on the gut microbiota remains limited, current findings indicate that excessive alcohol consumption compromises intestinal barrier function and induces dysbiosis (Engen et al., 2015). Cigarette smoking can alter gut microbial composition and diversity through mechanisms involving oxidative stress modulation, the disruption of intestinal tight junctions, and changes in mucin composition (Savin et al., 2018). Current studies report significant variations in donor cohort sizes for FMT, ranging from single donors to multi-donor cohorts (n=1–10) across published protocols (Lauko et al., 2023; Hsu et al., 2023; Hanske et al., 2009; Sánchez-Quintero et al., 2022; Crouzet et al., 2013; Salandre et al., 2023; Mao et al., 2021; Chiu et al., 2017; Xia et al., 2019; Sun et al., 2022; Liu et al., 2023; von Klitzing et al., 2017a; Ye et al., 2023; Zhang et al., 2013; Aluthge et al., 2020; Reygner et al., 2020; Fan et al., 2023; Renu et al., 2022; Zabolotneva et al., 2023; Cherbuy et al., 2019) (**Table 1**). Research from the Human Microbiome Project has confirmed that significant heterogeneity exists in gut microbial compositions and relative abundances between individuals, even among healthy populations (Human Microbiome Project Consortium, 2012a). Although single-donor FMT ensures traceable microbial origins, it does not adequately address population-level microbial diversity. Conversely, multi-donor strategies enhance ecological validity through sample pooling but increase operational complexity in terms of specimen collection and processing.

**Table 1:** Summary of methodological parameters for fecal sample cohort, collection, transport and storage in human microbiota-associated (HMA) studies.

Donor cohort sizes	Fecal collection methodologies	Time interval/storage conditions before processing	Fecal transport	Fecal storage		References
				Cryoprotective agents	Temperature	
A healthy adult donor		Stored at 4°C under anaerobic conditions and processed within 12 h				(Le Bihan et al., 2015)
3 patients with acute stroke		Frozen at −80°C immediately			−80°C	(Xia et al., 2019)
2 cohorts of 6 donors		≤2h			−80°C	(Liu et al., 2023)
5 healthy donors					−80°C	(von Klitzing et al., 2017a)
2 cohorts of 1 donors		Transferred to an anaerobic cabinet immediately		20% glycerol	−80°C	(Ye et al., 2023)
6 essential tremor patients and 6 healthy controls		≤4h		50% sterile glycerol	−80°C	(Zhang et al., 2023)
5			With dry ice		−80°C	(Van Den Ham et al., 2023)
4 female donors				50% glycerol	−80°C	(Aluthge et al., 2020)
6 healthy donors		≤2h		The maltodextrin-trehalose cocktail, 10% glycerol or 80% glycerol	−80°C	(Reygner et al., 2020)
3 females patients with anorexia nervosa and 3 healthy controls				20% glycerol	−80°C	(Fan et al., 2023)
5 obese and 5 healthy lean children	A sterile glass bottle			15% glycerol	−80°C	(Renu et al., 2022)
2 cohorts of 5 donors	A terile screwcap bottle with a sterile anaerobic medium	≤0.5h	With ice	15% glycerol	−80°C	(Dhakal et al., 2019)
10 adult human donors (5 males and 5 females) and a 3-mo old healthy baby	A completely filled airtight containers			10% glycerol	−80°C	(Zhang et al., 2013)
1		≤6h		10% sterile glycerol	–70°C	(Lauko et al., 2023)
10		≤2h		10% glycerol	–80°C	(Salandre et al., 2023)
A healthy male	An anaerobic box	≤1h				(Brandi et al., 2024)
	A sterile plastic cup	Storage at −20°C before processing		20% glycerol	–80°C	(Grabrucker et al., 2023)
	An stool sampler	Immediately transported to the laboratory and frozen at − 80 °C before processing.				(Liu et al., 2020)
4 female healthy donors	Paper sheets	Immediately transferred into sterilized containers, placed in an AnaeroPouch with a CO_2_ generator, and stored at −80°C before processing.				(Tamura et al., 2019)
3		Immediately transferred into an anaerobic chamber				(Liu et al., 2022)
		≤2h				(Sjöland et al., 2023)
3 constipated-predominant irritable bowel syndrome patients and 3 healthy controls	With Anaerocult A sachet	≤3h				(Gobert et al., 2016)
		≤2h		10% glycerol	−80°C	(Staley et al., 2017)
3	Sterile plastic boxes	Kept under anoxic conditions by using Anaerocult A and stored at 4°C for a maximum of 6 h before processing.				(Humblot et al., 2005)
		Stored at 4°C in an anaerobiosis generator within 24 h before processing			−80°C	(Spatz et al., 2023)
2 cohorts of 4 donors		Stored at 4°C before transport	With ice packs		−80 °C	(Glenny et al., 2021)
		Stored at −80°C immediately	In containers cooled by dry ice		−80°C	(Togao et al., 2023)
		Immediately frozen before being stored in liquid nitrogen within 15 min.			−80°C	(Nagao-Kitamoto et al., 2016)
5					−80°C	(von Klitzing et al., 2017b)
A female healthy donors		Stored at 4 °C in an anaerobiosis generator immediately, and processed within 24 h.			-80°C	(Wrzosek et al., 2018)
	Sterile tubes		Dry ice	30% glycerol	−80°C	(Feehley et al., 2019)
5 healthy donors					−80°C	(Heimesaat et al., 2019)
	Tubes without any additive	≤4h		20% glycerol	−80°C	(Tintelnot et al., 2023)
		Immediately frozen at -20°C	Frozen		−80°C	(Ridaura et al., 2013)
3				10% glycerol	−80°C	(Sánchez-Quintero et al., 2022)
2	Disposable coffee filter-like fecal collection devices	Immediately frozen at -80°C			−80°C	(Hintze et al., 2014)
2 cohorts of 3 donors				50% glycerol	−80°C	(Kaiser et al., 2021)
		Frozen at –80°C before processing		15% glycerol	−80°C	(Britton et al., 2019)

Optimal donor selection for microbiota studies requires stringent criteria. Based on the above evidence, we believe that healthy donors must demonstrate at least: (1) A ≥3-month abstinence from antibiotics, laxatives, and probiotic or prebiotic supplements (Le Bihan et al., 2015; Aluthge et al., 2020; Chung et al., 2012; Brandi et al., 2024; Gérard et al., 2004; Respondek et al., 2013; Zhang et al., 2023; Chen et al., 2020; Xia et al., 2019); (2) the absence of gastrointestinal disorders or active infections (Reygner et al., 2020; Tamura et al., 2019; Gobert et al., 2016; Saint-Cyr et al., 2013; Lauko et al., 2023; Nagao-Kitamoto et al., 2016, 2020; Salandre et al., 2023; Zabolotneva et al., 2023); (3) adherence to a nutritionally balanced diet (Cherbuy et al., 2019; Dong et al., 2021); (4) A preference for non-smokers and non-drinkers (Zabolotneva et al., 2023; Grabrucker et al., 2023; Gobert et al., 2016); and (5) compliance with fecal collection protocols. Disease cohort donors require additional validation that includes: (1) diagnostic confirmation per established clinical criteria (Zhang et al., 2023; Hsu et al., 2023; Fan et al., 2023; Demir et al., 2022; Zhang et al., 2020; Chen et al., 2020; Duan et al., 2019); (2) the exclusion of confounding comorbidities that could affect gut microbiota (Hsu et al., 2023; Fan et al., 2023; Chen et al., 2020; Duan et al., 2019; Xia et al., 2019); (3) the absence of active infectious diseases (Staley et al., 2017). Fecal samples could be initially collected from multiple donors, after which a suitable number of optimal and representative specimens could be selected for downstream experiments (Fan et al., 2023; Gobert et al., 2016; Xia et al., 2019; Zhang et al., 2023).

### 2.2 Fecal collection

#### 2.2.1 The time of fecal collection

Both humans and animals, along with their gut microbiotas, are affected by temporal rhythms. Research has demonstrated that 10% of the bacterial operational taxonomic units (OTUs) in humans and 15% of those in mice show significant circadian fluctuations in terms of relative abundance (Thaiss et al., 2014). Reitmeier et al. analyzed fecal samples from 1,943 participants with recorded collection times and revealed that 70% exhibited defecation patterns concentrated between the hours of 5:00–11:00 (Reitmeier et al., 2020). Throughout the day, distinct taxonomic groups dominate the gut microbiota. *Firmicutes* prevail during daylight hours, for example, whereas *Bacteroidetes* predominate nocturnally (Reitmeier et al., 2020). Current clinical FMT protocols lack standardized stool collection timing. In the preparation of animal models for FMT-based HMA animal model preparation, certain studies have utilized stool samples obtained from donors’ first morning bowel movements (Zhang et al., 2020; Mao et al., 2021; Sun et al., 2022).

#### 2.2.2 The methodology of fecal collection

Current methodologies for fecal sample collection exhibit significant heterogeneity. Certain protocols require donors to defecate directly into an anaerobic box (Brandi et al., 2024; Gobert et al., 2016), while others use sterile containers or specialized stool collection kits (Hintze et al., 2014; Grabrucker et al., 2023; Liu et al., 2020). Alternative approaches involve paper sheets and immediately transferring them into sterilized containers (Tamura et al., 2019). Standardized collection tools, exemplified by stool collection kits, present three primary advantages. First, they minimize oxygen exposure to protect anaerobic taxa. Second, they prevent environmental contamination, such as from toilet water and urine. Third, they enhance donor compliance through improved hygienic handling and sensory acceptability. The commode kit has gained widespread adoption in large-scale cohort studies such as the Human Microbiome Project, owing to its user-friendly design (Franzosa et al., 2014; Human Microbiome Project Consortium, 2012b). Despite achieving operational simplicity and cost optimization, these systems require detailed instructional protocols and incur additional research expenditures. Conversely, evidence demonstrates that paper-based collection methods preserve fecal microbial diversity and community structure without significant alteration (Al et al., 2018), offering a viable alternative for resource-constrained investigations.

#### 2.2.3 Time interval and storage conditions before processing

For fresh samples, the clinical FMT protocols emphasize that the primary recommendation is to process them within 6 h (Cammarota et al., 2017; Lopetuso et al., 2023). Fecal samples should be stored at a temperature of 20°C–30°C (Cammarota et al., 2017) or at ≤4°C prior to processing (Lopetuso et al., 2023). If feasible, anaerobic storage and processing should be utilized (Cammarota et al., 2017). Similar protocols apply to FMT-based HMA animal model preparation: in some studies, samples were required to be processed in an anaerobic chamber immediately after defecation (Tamura et al., 2019; Ye et al., 2023; Liu et al., 2022; Wrzosek et al., 2018). Consistent with clinical FMT protocols, some studies require microbial slurry extraction and FMT administration to be completed within 2–6 hours post-collection (Sjöland et al., 2023; Gobert et al., 2016; Staley et al., 2017; Zhang et al., 2023; Lauko et al., 2023). When immediate processing is unfeasible, stool samples were stored under anoxic conditions at 4°C for a maximum 6–24 h (Humblot et al., 2005; Wrzosek et al., 2018; Le Bihan et al., 2015; Spatz et al., 2023)(**Table 1**). These preservation measures aim to maintain donor microbial viability (MV) and protect obligate anaerobes, which outnumber aerobic bacteria by 100–1000× in the human gut (Widjaja and Rietjens, 2023). Lower anaerobe abundance has been reported to correlate with dysbiosis-associated pathologies such as irritable bowel syndrome (IBS) and inflammatory bowel disease (IBD) (Rajilić-Stojanović et al., 2011; Sokol et al., 2009). Insufficient anaerobic protection may therefore compromise experimental outcomes through microbial community variation. However, clinical evidence demonstrates comparable efficacy between anaerobic and aerobic FMT preparations when treating *Clostridioides difficile* infections (Lee et al., 2016). This equivalence may stem from spore-forming bacterial genera, which constitute 50–60% of healthy gut microbiota and exhibit oxygen-resistant sporulation capabilities and thus facilitate inter-host transmission (Browne et al., 2016).

In summary, it is imperative for researchers to meticulously record the precise defecation times of participants and to prioritize the collection of fecal samples from the same timeframe in order to mitigate potential confounding variables associated with circadian rhythms (Thaiss et al., 2014; Reitmeier et al., 2020; Zhang et al., 2020; Mao et al., 2021; Sun et al., 2022). The optimal collection methodology should be selected based on donor cohort size and degree of cooperation, with a standardized sampling methodology maintained to minimize technical variability. Ideally, fresh fecal samples should be processed within 2 h of collection, with a maximum allowable delay of 6 h (Sjöland et al., 2023; Gobert et al., 2016; Staley et al., 2017; Zhang et al., 2023; Lauko et al., 2023). In instances where immediate processing is not feasible, it is advisable to refrigerate the samples at 4°C (Humblot et al., 2005; Spatz et al., 2023). The adoption of anaerobic preservation and processing protocols should be guided by the available laboratory resources and the specific aims of the research. These findings provide preliminary insights into fecal collection and processing methods, but further research is needed for validation.

### 2.3 Fecal transport and storage protocols

The standardized handling of fresh fecal samples requires predefined transport and storage solutions when immediate processing is not feasible. Current methodologies demonstrate variations in the transportation and preservation of stool samples(**Table 1**): some studies advocate for ice-based transportation without defined temperature parameters (Dhakal et al., 2019; Glenny et al., 2021), while others recommend using dry ice for cryopreservation prior to shipping (Van Den Ham et al., 2023; Togao et al., 2023; Feehley et al., 2019). A broad consensus exists among researchers regarding –80°C as the optimal long-term storage temperature for fecal specimens (Nagao-Kitamoto et al., 2016; von Klitzing et al., 2017b; Wrzosek et al., 2018; Feehley et al., 2019; Heimesaat et al., 2019; Spatz et al., 2023; Tintelnot et al., 2023; Togao et al., 2023; Zhang et al., 2023; Xia et al., 2019; Liu et al., 2023; Ye et al., 2023; Zhang et al., 2023; Van Den Ham et al., 2023; Aluthge et al., 2020; Reygner et al., 2020; Fan et al., 2023; Renu et al., 2022). Although pragmatic protocols permit short-term preservation at –20°C before inoculum preparation (Grabrucker et al., 2023; Ridaura et al., 2013). Alternatively, storage and transportation at 4°C is permitted within a strict ≤24 h limit (Spatz et al., 2023; Wrzosek et al., 2018).

Current research has not explored how different storage conditions of fecal samples may influence the outcomes of FMT. Nevertheless, multiple studies have reported the finite effects of storage conditions on fecal microbiota. Fouhy et al. observed no significant compositional differences between fresh, dry ice flash-frozen, and –80°C-stored (for 7 days) fecal samples (Fouhy et al., 2015). Tedjo et al. confirmed microbiota stability following 24 h storage at 4°C, and 1-week storage at –20°C preservation whether for healthy, IBS, and IBD cohorts (Tedjo et al., 2015). Similarly, Choo et al. demonstrated that healthy donor fecal samples stored at 4°C for 72 h exhibited no statistically significant differences regarding microbial composition and diversity compared to their –80°C cryopreserved counterparts (Choo et al., 2015). Therefore, 4°C refrigeration and –20°C freezing are recommended as short-term transportation and preservation conditions, while –80°C cryopreservation is reserved for long - term storage.

In the context of cryopreservation, methods encompass direct freezing (Ridaura et al., 2013) as well as the incorporation of various cryoprotective agents like 10–50% glycerol solutions (Lauko et al., 2023; Ye et al., 2023; Feehley et al., 2019; Sánchez-Quintero et al., 2022; Aluthge et al., 2020; Zhang et al., 2023; Fan et al., 2023; Renu et al., 2022; Dhakal et al., 2019; Zhang et al., 2013; Lauko et al., 2023; Salandre et al., 2023; Grabrucker et al., 2023; Feehley et al., 2019; Tintelnot et al., 2023; Kaiser et al., 2021).The academic community remains divided concerning cryoprotectant. Advocates posit that freeze-thaw cycles (FTCs) compromise bacterial viability (Postgate and Hunter, 1961), necessitating the use of protective agents. Due to the uncertainties surrounding the effects of glycerol’s cellular permeation on bacterial viability, novel formulations such as maltodextrin-trehalose have been developed. The maltodextrin-trehalose have been validated through multi-phase assays to optimally preserve fecal microbial vitality during both freezing and thawing (Burz et al., 2019; Reygner et al., 2020). Conversely, some researchers suggest that direct ultra-low-temperature (−80°C) preservation without additives can maintain microbial composition without significant alteration (Tedjo et al., 2015). Three clinical studies provide evidence that the therapeutic effects of fresh and cryopreserved FMT preparations are comparable (Lee et al., 2016; Satokari et al., 2015; Sintes et al., 2024). However, a comparative trial indicated that fecal samples frozen without cryoprotectants showed changes in composition, viability, and cultivability upon thawing compared to fresh feces (Bilinski et al., 2022). Therefore, cryopreservation method should consider the use of cryoprotectants to maintain MV and composition, especially when samples undergo multiple FTCs. For short-term fecal sample storage, direct freezing at ultra-low temperatures without additives may be sufficient for preserving microbial integrity in certain contexts.

### 2.4 Fecal suspensions preparation

Fresh fecal specimens are typically reconstituted using phosphate-buffered saline (PBS) (Mao et al., 2021; Chiu et al., 2017; Dong et al., 2021; Sun et al., 2022; Sánchez-Quintero et al., 2022, 2023; Wahlström et al., 2017) or brain heart infusion (BHI) culture medium (Spatz et al., 2023; Wrzosek et al., 2018) before FMT administration, as shown in **Table 2**. During the post-thaw processing of cryopreserved fecal samples, common dilution vehicles include sterile saline (Hintze et al., 2014; Huang et al., 2020; Van Den Ham et al., 2023; Zabolotneva et al., 2023), PBS buffer (Grabrucker et al., 2023; Kaiser et al., 2021; Xia et al., 2019; Ye et al., 2023), media contain glycerol (Britton et al., 2019), and BHI medium (Tintelnot et al., 2023). The standard dilution ratios range from 1:10 to 1:1000 (w/v) (Le Bihan et al., 2015; Nagao-Kitamoto et al., 2016; Wrzosek et al., 2018; Crouzet et al., 2013; Togao et al., 2023; Zabolotneva et al., 2023; Wahlström et al., 2017)(**Table 2**). Sample preparation strategies include donor-specific retention through individual processing (Nagao-Kitamoto et al., 2016) and homogenized aliquots via pooled sample blending (Sánchez-Quintero et al., 2023). Clinical guidelines explicitly discourage the pooling of fecal samples from multiple donors during processing, to maintain donor traceability and mitigate the potential for pathogen dissemination (Keller et al., 2021). However, HMA model development strategies often involve compositing donor material to achieve a uniform distribution of human-derived gut microbiota across recipient animals (Sánchez-Quintero et al., 2023), thereby minimizing inter-individual variability. Fecal homogenization tools include traditional mortar-pestle grinding (Turnbaugh et al., 2009), dedicated blenders (Lauko et al., 2023; Reygner et al., 2020), the Ultra-Turrax blender (Humblot et al., 2005), and the Nanogenizer-Titanium High-Pressure Homogenizer (Staley et al., 2017; Kaiser et al., 2021). At present, there is a deficiency of comparative research examining the effects of various homogenization instruments on FMT. The available evidence suggests that following the blending process using either a blender or a pneumatic mixer, high-throughput DNA sequencing reveals a notable decrease in intra-sample heterogeneity (Hsieh et al., 2016). Dilution and filtration are common procedures during suspension preparation (Grabrucker et al., 2023; Zabolotneva et al., 2023; Zhang et al., 2023; Han et al., 2021; Reygner et al., 2020; Zhang et al., 2020; Staley et al., 2017; Zhang et al., 2013), which may help remove food debris, reduce the viscosity of the suspension, and prevent catheter occlusion during administration. Drawing from the aforementioned information, we recommend blending fecal samples followed by sequential dilution, homogenization, and filtration to obtain representative suspensions. Researchers should explicitly document their procedural details during such experiments—particularly the diluent composition and dilution ratio—to enhance experimental reproducibility.

**Table 2:** Preparation and storage conditions of fecal suspensions.

Fecal sample		Condition	Dilution		Storage	References
Fresh/frozen	Dosage(g)		Solution	Concentration		
Fresh	1	Anaerobic	Anaerobic mineral solution containing 5 g/l NaCl, 2 g/l glucose and 0.3 g/l cysteine–HCl	1:10(wt:v)		(Le Bihan et al., 2015)
Fresh		Anaerobic	Anaerobic mineral solution	1:1000(wt:v)		(Crouzet et al., 2013)
Fresh			0.1 M phosphate-buffered saline (PBS) buffer (pH 7.2)			(Mao et al., 2021)
Fresh		Anaerobic	0.85% saline	1:50(wt:v)		(Hanske et al., 2009)
Frozen			Sterile PBS containing 20% glycerol	100 mg/mL	−80°C	(Grabrucker et al., 2023)
Frozen			Sterile saline	100 mg/mL		(Hintze et al., 2014)
Fresh	1		PBS	1:9		(Chiu et al., 2017)
Frozen	0.2-0.5	Anaerobic	Anaerobic Mega Media	100 mg/mL		(Hutchison et al., 2024)
	1	Anaerobic	LuriaBertani medium containing 15% glycerol	1g:30 mL		(Hsu et al., 2023)
Frozen			Sterile PBS			(Xia et al., 2019)
Fresh			PBS	1:9(wt:v)		(Sun et al., 2022)
Fresh		Anaerobic	Brain-Heart Infusion (BHI) supplemented with 0.5 mg/mL L-cysteine and 20% skim milk	1:100(wt:v)	−80°C	(Spatz et al., 2023)
	0.5		PBS buffer containing 0.5 g/L cysteine	100 mg/mL		(Liu et al., 2023)
Fresh			BHI supplemented with 0.5 mg/mL L-cysteine and 20% skim milk (vol/vol)	1:100(wt:v)	−80 °C	(Wrzosek et al., 2018)
Frozen			Sterile PBS		−80 °C	(von Klitzing et al., 2017b, 2017a)
Frozen			Sterile saline			(Huang et al., 2020)
Fresh		Anaerobic	Sterile PBS with 20% glycerol		−80°C	(Ye et al., 2023)
Frozen			BHI			(Tintelnot et al., 2023)
Fresh			Saline with 50% sterile glycerol		−80°C	(Zhang et al., 2023)
Frozen			Sterile saline	1:10(wt:v)		(Van Den Ham et al., 2023)
	0.1		Sterile PBS	1g:15mL		(Lin et al., 2021)
	1		Saline	1:10(wt:v)		(Togao et al., 2023)
Frozen	0.1		Saline	1:10(wt:v)		(Zabolotneva et al., 2023)
Frozen	2.5	Anaerobic	Sterile Similac^®^ infant formula	1g:20mL	−80°C	(Aluthge et al., 2020)
Frozen			The maltodextrin-trehalose cocktail, 10% glycerol or 80% glycerol	1:6 (v:v)	−80°C	(Reygner et al., 2020)
Fresh			Sterile PBS	1:10 (v:v)	−80°C	(Sánchez-Quintero et al., 2023, 2022)
Fresh	50		Sterile normal saline, 0.1 M PBS containing 10% sterile medical glycerin	1:50(wt:v)	−80 °C	(Han et al., 2021; Zhang et al., 2020)
Frozen	10	Anaerobic	0.1 M PBS	1:10(wt:v)		(Dong et al., 2021)
Fresh		Anaerobic	Sterile pre-reduced PBS			(Liu et al., 2022)
Fresh	0.25	Anaerobic	LYBHI medium (containing 0.05% cysteine and 0.2% hemin) with 20% glycerol		−80 °C	(Fan et al., 2023)
Fresh	0.5		PBS	1:10		(Wahlström et al., 2017)
Fresh			0.1 M PBS with 15% glycerol		−80°C	(Renu et al., 2022)
Fresh			Sterile glycerol 15% (v/v)		−80°C	(Dhakal et al., 2019)
Fresh			Reduced PBS containing 10% glycerol	1:10	−80°C	(Zhang et al., 2013)
Fresh			PBS containing 10% glycerol		−80°C	(Staley et al., 2017)

### 2.5 Fecal microbiota assessment methodologies

Before FMT implementation, fecal suspensions are typically assessed via culturing-based methods (Bereswill et al., 2011), flow cytometry (Bilinski et al., 2020), 16S rRNA sequencing (Bereswill et al., 2011), shotgun metagenomic sequencing (Liu et al., 2023), or agar spot assays (Reygner et al., 2020). These analytical modalities collectively evaluate MV, composition, quantitation, and antagonistic capacity against specific bacterial strains. Conventional culturing methods typically detect only ~30–50% of viable gut microbes (Adak and Khan, 2019). Bilinski et al. demonstrated that flow cytometry with fluorochromes provides superior bacterial viability validation (Bilinski et al., 2020). The next-generation sequencing (NGS)—including 16S rRNA gene sequencing and shotgun metagenomics represent the common methodologies used in microbial studies, both of which carry distinct advantages. The 16S rRNA gene sequencing is well-suited to large-scale cohort analyses. However, it suffers from reduced accuracy in terms of species-level classification and functional profiling capacity—thus precluding the detection of strain-level variations (Jovel et al., 2016; Wensel et al., 2022). Conversely, the shotgun metagenomics facilitates strain identification and functional prediction but carries substantially higher costs (Jovel et al., 2016; Wensel et al., 2022). The agar spot test serves as a simple and effective preliminary screening tool for selecting antagonistic fecal samples in FMT-bacterial infection therapy, thus reducing downstream experimental expenditures (Salandre et al., 2023). In summary, shotgun metagenomic sequencing and agar spot assays are considered more suitable analytical methods for conducting detailed characterizations of specific bacterial strains or for selecting functionally specialized samples. However, the viable microbial number and 16S rRNA gene sequencing are recommended for initial community profiling due to its cost-effectiveness, ease of use, and suitability for large-scale or routine analyses.

## 3 Recipient selection

### 3.1 Recipient types

#### 3.1.1 Germ-free animals

Germ-free (GF) animals are born and maintained in isolators throughout their lifespans, thus having minimal or no microbial exposure (Dremova et al., 2023). GF mice are still the most extensively used model organisms of this type—although axenic pig, dog, and chicken systems have been successfully generated through the progressive development of various technologies (Dhakal et al., 2019; Uzbay, 2019; Zhang et al., 2013). The establishment of gnotobiotic models through the colonization of GF animals with defined microbial consortia can provide controllable platforms for investigating host-microbe interactions (Dremova et al., 2023). Excluding the confounding effects of indigenous microbiota and antibiotics, this approach is widely regarded as an optimal strategy for generating human microbiota-associated (HMA) models. The applications of GF animals primarily include the following aspects: (1) elucidating the relationship between microbes and diseases to explore pathogenic mechanisms (Huang et al., 2020); (2) investigating the protective roles of microbes, such as resistance to the pathogen *Clostridioides difficile* (Sulaiman et al., 2025, 2024), mitigation of obesity (Mao et al., 2021), and alleviation of gastrointestinal discomfort (Rocha Martin et al., 2022); (3) studying metabolites produced by gut microbial communities, such as short-chain fatty acids (Liu et al., 2025), bile acids (Xue et al., 2025), and lactate (Li et al., 2022); (4) examining factors influencing microbial communities, including responses and functional outputs to dietary fibers and different types of diets (Feng et al., 2022; Turnbaugh et al., 2009); and (5) exploring the mechanisms by which drugs target the gut microbiota for therapeutic effects (Li et al., 2023). However, the utility of axenic models is constrained by three intrinsic barriers: first, the operational costs of isolator-based husbandry and sterile maintenance are prohibitive (Kennedy et al., 2018); second, open-environment behavioral assays and coinfection studies cannot be implemented (Kennedy et al., 2018); and third, immuno-developmental deficits inevitably arise because of the absence of gut microbiota (Kennedy et al., 2018). Collectively, these limitations have reduced the applicability of such models in terms of sophisticated pathophysiological research.

#### 3.1.2 Altered Schaedler’s flora animals

To circumvent the immunological and developmental deficits of GF animals while maintaining controlled microbial status, altered Schaedler’s flora (ASF) animals were developed as well-defined microbiota models. Originating from Schaedler’s 1965 longitudinal tracking of gut microbiota succession in Nelson-Collins Swiss mice from birth to weaning, this model incorporates a standardized bacterial consortium that has been designated Schaedler’s flora (Schaedler et al., 1965). In 1978, Orcutt et al. refined and standardized this microbial consortium for stable intestinal colonization in murine hosts, and formally designated it ASF (Trexler and Orcutt, 1999). ASF serves as a representation of conventional murine gut microbiota (Deloris Alexander et al., 2006), demonstrating heritable stability through transgenerational propagation after colonization (Sarma-Rupavtarm et al., 2004). Compared to GF mice, ASF mice exhibit normal gastrointestinal architecture and physiological functions, along with fully developed immune systems (Proctor et al., 2022; Sarma-Rupavtarm et al., 2004). These animals are preferentially used to investigate specific microbial influences, intestinal mucosal responses, and the development of the enteric nervous system (Wymore Brand et al., 2015). However, the use of ASF mice in HMA studies remains scarce. Staley et al. demonstrated separate human donor microbiota transferability to ASF mice but revealed divergent outcomes. One cage exhibited significant microbial divergence from the donors (*P*=0.002), while another maintained no detectable divergence (*P*=0.012) (Staley et al., 2017). This heterogeneity suggests a potential niche competition between native ASF and humanized microbiomes (Staley et al., 2017), though the specific mechanisms underlying this phenomenon merit further investigation. The current evidence in the field is insufficient in terms of clearly defining the utility of ASF systems regarding humanized microbiota transfer, thus demanding expanded experimental validation.

#### 3.1.3 Antibiotic administration-induced pseudo-germ-free animals

Although rodent and human gut microbiomes share taxonomic similarities, 85% of the microbial genera present in rodents are absent in humans (Ley et al., 2005). Thus, pre-FMT preparation must maximize the depletion of native microbiota to enhance the engraftment efficiency of transplanted communities. Specific pathogen-free (SPF) animals are those maintained in barrier-controlled environments, with certification confirming the absence of a defined set of common pathogens to which the species is typically exposed in a natural setting (Dobson et al., 2019; Lane-Petter, 1962). The establishment of pseudo-GF animals using various antibiotic regimens (**Table 3**) constitutes the primary preparatory phase for establishing HMAs based on SPF animals. This strategy originated in 1954 with Bohnhoff’s seminal discovery that the oral administration of high-dose streptomycin (50 mg) significantly increased the susceptibility to *Salmonella enteritidis* infection in mice (Bohnhoff et al., 1954). This discovery revealed that antibiotics disrupt gut microbiota homeostasis. It also established an experimental approach that leverages the antimicrobial suppression of native microbiota to enhance colonization potential. Subsequent studies demonstrated a 10× reduction in fecal 16S rDNA load and drastic structural alterations in microbial communities by day 10 of antibiotic treatment (Hill et al., 2010). This significantly increased the probability of effective donor microbiota colonization via FMT.

**Table 3:** Exemplary intestinal preparation strategies for recipient cohorts.

Administration techniques	Gut decontamination Strategy	Add-ons	Detection methods and depletion status of intestinal microbiome	Antibiotic washout period	References
Oral gavage	Vancomycin (100 mg/kg), Neomycin Sulfate (200 mg/kg), Metronidazole (200 mg/kg), and Ampicillin (200 mg/kg), Qd, 5 consecutive days.		16S rRNA gene sequencing		(Chen et al., 2020; Kong et al., 2023)
	Vancomycin (100 mg/kg), Neomycin Sulfate (200 mg/kg), Metronidazole (200 mg/kg), and Ampicillin (200 mg/kg), Qd, 3 consecutive weeks.		16S rRNA gene sequencing		(Liang et al., 2020)
	Vancomycin (400 mg/kg), Neomycin (400 mg/kg), and Metronidazole (200 mg/kg), 3 consecutive days.		16S rRNA gene sequencing		(Sun et al., 2022)
	Vancomycin (10 g/L), Metronidazole (20 g/L), Gentamicin (4 g/L), and Ampicillin (20 g/L), 0.2mL/Qd, 3 consecutive days.				(Xia et al., 2019)
Oral gavage+ subcutaneous injection	Amoksiklav (2 × 457 mg/5 mL) 0.2 mL/d + Ciprinol con infusion (5 × 10 mL/100 mg), 0.1 mL/Q12h, 5 consecutive days.				(Lauko et al., 2023)
*Ad libitum* antibiotic solution	Phase 1: Ertapenem Sodium (1 g/L), Neomycin Sulfate (1 g/L), and Vancomycin Hydrochloride (1 g/L) administered for 7 consecutive days; Transition: Standard drinking water *ad libitum* for 2 days; Phase 2: Ampicillin (1 g/L), Cefoperazone Sodium salt (1 g/L), and Clindamycin Hydrochloride (1 g/L) administered for 7 days; Transition: Standard drinking water *ad libitum* for 2 days; Phase 3: Repeat phase 1 for 7 consecutive days.			48h	(Kaiser et al., 2021)
	Amoxicillin (0.5 g/L) for 8 consecutive days.			24h	(Salandre et al., 2023)
	Ampicillin (1 g/L), Vancomycin (500 mg/L), Ciprofloxacin HCL (200 mg/L), and Imipenem (250 mg/L) for 7 consecutive days		16S rRNA gene sequencing	72h	(Grabrucker et al., 2023)
	Ampicillin (1 g/L), Vancomycin (500 mg/L), Ciprofloxacin (200 mg/L), Imipenem (250 mg/L) and Metronidazole(1 g/L) for 6 consecutive weeks		16S rRNA gene sequencing confirmed bacterial absence in the generated secondary abiotic mice fecal samples	72h	(Heimesaat et al., 2018)
	Ampicillin (1 g/L), Vancomycin (500 mg/L), Ciprofloxacin (200 mg/l), Imipenem (250 mg/L) and Metronidazole(1 g/L) for 6–8 consecutive weeks				(Bereswill et al., 2011)
	Ampicillin (1 g/L), Vancomycin (500 mg/L), Neomycin (100 mg/l) and Metronidazole (1 g/L) for 6 consecutive weeks	10% sucrose	Bacterial culture, Quantitative PCR, and Fluorescent *in-situ* Hybridization (FISH): 96% stool DNA reduction at day 3 of antibiotic treatment compared to baseline (*P* < 0.00001).		(Amorim et al., 2022)
	Ampicillin (2 g/L) plus Sulbactam (1 g/L) for 8 consecutive weeks.			48h	(Heimesaat et al., 2024; Shayya et al., 2023)
	Ampicillin plus sulbactam (1 g/L), Vancomycin (500 mg/L), Ciprofloxacin (200 mg/L), Imipenem (250 mg/L), and Metronidazole (1 g/L) for 6–8 consecutive weeks.			72h	(von Klitzing et al., 2017b, 2017a)
	Ampicillin plus sulbactam (1 g/L), Vancomycin (500 mg/L), Ciprofloxacin (200 mg/L), Imipenem (250 mg/L) and Metronidazole (1 g/L) for 8 consecutive weeks.			72h	(Kløve et al., 2020)
	Ampicillin (1 g/L), Neomycin Sulfate (1 g/L), Metronidazole (1 g/L), and Vancomycin Hydrochloride (1 g/L) for 4 consecutive weeks.			48h	(Zhan et al., 2024)
	Ampicillin (1 g/L), Cefoperazone Sodium salt (1 g/L), and Clindamycin Hydrochloride (1 g/L) administered for 7 days.		16S rRNA gene sequencing: antibiotic treatments significantly reduced Shannon community diversity indices relative to those before antibiotic exposure or among donor samples (Tukey’s *post hoc* test *P* < 0.0001).	48h	(Staley et al., 2017)
	Ampicillin (1 g/L), Vancomycin (500 mg/L), Neomycin (500 mg/L), Gentamicin (100 mg/L) and Erythromycin (10 mg/L) for 2 consecutive weeks.			48h	(Zhang et al., 2023)
	Ampicillin (1 g/L), Neomycin (1 g/L), Metronidazole (1 g/L) and Vancomycin Hydrochloride (1 g/L) administered for 7 days.			96h	(Liu et al., 2023)
	Ciprofloxacin (30 mg/kg) administered for 4 days.		16S rRNA gene sequencing: antibiotic treatment reduced the mouse’s autochthonous gut microbial load by 1–2 orders of magnitude.	3h	(Wos-Oxley et al., 2012)
*Ad libitum* antibiotic solution + Intraperitoneal injection	Phase 1: Drinking water containing Kanamycin (0.4 mg/mL), Gentamicin (0.035 mg/mL), Colistin (850 U/mL), Metronidazole (0.215 mg/mL), and Vancomycin (0.045 mg/mL) for 3 consecutive days Washout: Standard water ad libitum for 1 day; Phase 2: Single intraperitoneal injection of Clindamycin (10 mg/kg).				(Reygner et al., 2020)
*Ad libitum* antibiotic solution+ Oral gavage	Phase 1: Drinking water containing Ampicillin (1 g/L) During the period; Phase 2: Orally gavage Vancomycin (5 mg/mL), Neomycin (10 mg/mL), and Metronidazole (10 mg/mL), 10 mL/kg/Q12h for 10 consecutive days.				(Sánchez-Quintero et al., 2022)
	Phase 1: Drinking water containing Ampicillin (1 g/L) During the period; Phase 2: Orally gavage Amphotericin B (1 mg/kg), Q12h for 3 consecutive days; Phase 3: Orally gavage Vancomycin (500 mg/L), Neomycin (100 mg/l), and Metronidazole(1 g/L)and Amphotericin B (1 mg/kg), Q12h for 14 consecutive days.			12h	(Hintze et al., 2014; Kim et al., 2024)
Oral gavage	After 1-hour fasting, oral gavage administration of polyethylene glycol 4000 (PEG4000)			4h	(Spatz et al., 2023)
	After 1-hour fasting, 200 μL of polyethylene glycol 4000 (PEG4000; 425 g/L) was administered via oral gavage at 20-minute intervals, with the cycle repeated 2–6 times.		Real-time qPCR analysis of the 16S rRNA gene sequencing: a significant 1-Log decrease (90% of the total bacteria), and reaching the plateau phase.		(Wrzosek et al., 2018)

The administration routes include *ad libitum* antibiotic solution (Amorim et al., 2022; Bereswill et al., 2011; Grabrucker et al., 2023; Heimesaat et al., 2024, 2018; Kaiser et al., 2021; Salandre et al., 2023; Shayya et al., 2023), oral gavage (Chen et al., 2020; Kong et al., 2023; Liang et al., 2020; Sun et al., 2022; Xia et al., 2019), and injection (Lauko et al., 2023). Among these, drinking antibiotic solutions offers maximal technical simplicity.However, it carries a risk of dehydration, which may result from animals avoiding water due to the taste of the antibiotic or from antibiotic-associated diarrhea caused by prolonged exposure to the solution (Hill et al., 2010; Reikvam et al., 2011; Xu et al., 2023). Modified regimens, such as removing gentamicin or supplementing with sweeteners, have failed to mitigate this issue (Hill et al., 2010; Reikvam et al., 2011). By contrast, gavage delivery circumvents the dehydration trap while displaying microbiota depletion-associated phenotypes (Hill et al., 2010; Reikvam et al., 2011).Furthermore, several investigators have combined various delivery modalities like “oral gavage + subcutaneous injection” (Lauko et al., 2023), “*ad libitum*antibiotic solution + intraperitoneal injection” (Reygner et al., 2020), and “*ad libitum*antibiotic solution + oral gavage” (Sánchez-Quintero et al., 2022) to achieve superior methodological outcomes.

Different antimicrobial agents exhibit different targeting mechanisms. Metronidazole selectively impacts anaerobes, vancomycin targets gram-positive bacteria, and ampicillin and ciprofloxacin act against both gram-positive and gram-negative species (Schubert et al., 2015; Zackular et al., 2016). Consequently, antibiotic cocktails (including dual or multiple antibiotics and antifungals) are essential for comprehensive microbial eradication (von Klitzing et al., 2017b, 2017a; Zhan et al., 2024).

In pseudo-GF animal models generation, different types of antibiotics exhibit varying dosages depending on the administration route. For example, the commonly used oral gavage dose of vancomycin is 100 mg/kg (Chen et al., 2020; Kong et al., 2023; Liang et al., 2020), while the dose via drinking water is 500 mg/L (Grabrucker et al., 2023; Amorim et al., 2022; Kløve et al., 2020; Heimesaat et al., 2018; von Klitzing et al., 2017b, 2017a; Bereswill et al., 2011). The typical gavage dose of ampicillin is 200 mg/kg (Kong et al., 2023; Chen et al., 2020; Liang et al., 2020), whereas the dose in drinking water is 1 g/L (Grabrucker et al., 2023; Amorim et al., 2022; Kaiser et al., 2021; Kløve et al., 2020; Heimesaat et al., 2018; von Klitzing et al., 2017b, 2017a; Bereswill et al., 2011). For metronidazole, the gavage dose is 200 mg/kg (Kong et al., 2023; Sun et al., 2022; Chen et al., 2020; Liang et al., 2020), while the drinking water concentration is 1 g/L (Amorim et al., 2022; Kløve et al., 2020; Heimesaat et al., 2018; von Klitzing et al., 2017b, 2017a; Bereswill et al., 2011). Treatment timeframes also vary significantly. Gavage persists for 3–21 days (Liang et al., 2020; Sun et al., 2022), whereas aqueous delivery via the drinking of antibiotic solutions lasts between 3–56 days (Heimesaat et al., 2024, 2018; Liu et al., 2023; Reygner et al., 2020; Shayya et al., 2023; Wos-Oxley et al., 2012). Amorim et al. administered broad-spectrum antibiotics (ampicillin 1 g/L, vancomycin 0.5 g/L, neomycin 1 g/L, and metronidazole 1 g/L) through drinking an antibiotic solution and subsequently quantified the depletion of gut microbiota (Amorim et al., 2022). They demonstrated a 96% reduction by day 3, progressive declines through days 7–14, and stabilization by day 21 (Amorim et al., 2022). Tirelle et al. compared administration routes across temporal fecal bacterial density profiles and reported that twice-daily gavage (amphotericin-B 0.1 g/L, ampicillin 10 g/L, neomycin trisulfate salt hydrate 10 g/L, metronidazole 10 g/L, and vancomycin hydrochloride 5 g/L) achieved a bacterial depletion efficiency comparable to that of drinking water (amphotericin-B 0.01 g/L, ampicillin 1 g/L, neomycin trisulfate salt hydrate 1 g/L, metronidazole 1 g/L, and vancomycin hydrochloride 0.5 g/L). They demonstrated significant depletion by day 4, which was sustained until day 12 without additional clearance effects (Tirelle et al., 2020). These findings indicate that 3-day administration achieves fundamental microbiota eradication regardless of the delivery method, whereas optimized durations maintain persistent effects. Prolonged treatment regimens risk inducing antibiotic-resistant strains and compromising the health of the model animals, altering their phenotypes and increasing their mortality rates (Hill et al., 2010; Tirelle et al., 2020).

Additionally, animal studies from rat donors have demonstrated that transplantation of homologous microbiota on the second day following antibiotic administration leads to novel microbial reorganization (Manichanh et al., 2010). This phenomenon may be attributed to collateral damage caused by antibiotic residues, which can adversely affect both native and transplanted microbial communities (Manichanh et al., 2010). Therefore, restoring sterile water for a certain period prior to FMT could help mitigate the interference caused by antibiotic residues. According to existing evidence, this period typically ranges from 48 to 72 hours (Grabrucker et al., 2023; Heimesaat et al., 2024, 2018; Kaiser et al., 2021; Kløve et al., 2020; Shayya et al., 2023; Staley et al., 2017; von Klitzing et al., 2017b, 2017a; Zhan et al., 2024; Zhang et al., 2023).

#### 3.1.4 Bowel cleansing-induced pseudo-germ-free animals

Laxative-based bowel-cleansing agents provide another effective microbiota-depleting strategy. Polyethylene glycol (PEG), a standard colonic preparation agent for colonoscopy procedures, has been used in many clinical studies to reduce microbial biomass and diversity when administered via split-dose regimens (Harrell et al., 2012; Jalanka et al., 2015; Zhou et al., 2025). Current clinical FMT guidelines rank PEG enemas as the optimal secondary preparatory intervention following antibiotic pretreatment (Cammarota et al., 2017). Wrzosek et al. demonstrated the applicability of PEG in animal bowel preparation protocols (Wrzosek et al., 2018). Murine models that received four cycles of intragastric 425 g/L PEG 4000 (200 µL per dose at 20 min intervals) achieved complete gastrointestinal evacuation with 90% reductions in microbial biomass (Wrzosek et al., 2018). Experimental data from mouse donor studies also indicated that 4-week regimens of PEG 400 or PEG 4000 (40% concentration, 100 µL oral gavage delivered 5 times weekly) significantly reduced gut microbial diversity in mice, with the 40% PEG 4000 cohort showing superior efficacy (Ishibashi et al., 2023). This approach preserves intestinal immune function and gut microbiome stability vs antibiotic-mediated depletion protocols (Wrzosek et al., 2018). In complex HMA models that require concurrent antibiotic therapy because of coinfection (Spatz et al., 2023), PEG lavage prevents antibiotic-associated carryover effects. However, as an osmotic cathartic, PEG requires elevated concentrations and substantial dosages to achieve effective intestinal clearance (Ishibashi et al., 2023; Le Roy et al., 2018)—which can induce electrolyte disturbances and dehydration. PEG-induced osmotic diarrhea disrupts the protective colonic mucus barrier, potentially influencing host immunocompetence (Tropini et al., 2018).

Overall, since antibiotic administration and bowel cleansing-induced pseudo-germ-free animals both retain residual native microbiota, these microbes may compete with the transplanted microbes or potentially develop into new ecological structures. Such models may not accurately represent a truly germ-free environment (Amorim et al., 2022; Hill et al., 2010; Tirelle et al., 2020; Wrzosek et al., 2018). Therefore, GF animals may be the optimal research model for exploring the causal relationships between microbiota and phenotypes. However, antibiotic-mediated pseudo-axenic models exhibit methodological superiority in studies focused on immunological regulation, developmental research, or targeted pathogen challenges. PEG bowel-cleansing protocols merit primary consideration if required to circumvent antibiotic-induced microbiota remodeling or residual impacts.

### 3.2 Recipient age

Human microbiota-associated (HMA) animal models common receptor types and ages include: (1) Fischer 344 rat, 8 -week-old (Crouzet et al., 2013); (2) Sprague dawley (SD) Rat, with various starting ages including 8 -week-old (Mao et al., 2021), 10 -week-old (Hanske et al., 2009), and 13-week-old (Grabrucker et al., 2023); (3) C57BL/6 mouse, with a range of starting ages from 3 to 8-week-old (Chiu et al., 2017; Hutchison et al., 2024; Hsu et al., 2023; Xia et al., 2019; Sun et al., 2022; Spatz et al., 2023; Salandre et al., 2023; Liu et al., 2023; Wrzosek et al., 2018; Huang et al., 2020; von Klitzing et al., 2017a); (4) BALB/c mouse, with various starting ages including 4,6,8-week-old (Kibe et al., 2005; Lin et al., 2021; Togao et al., 2023; Zabolotneva et al., 2023); as shown in **Table 4**. Due to the lack of humanized microbiota animal studies across different age groups, a study describing FMT from animal donors to same-species recipients of varying ages was selected as an indirect reference for analysis. In this study, age significantly influenced the efficacy of gut microbiota colonization (Le Roy et al., 2018).Comparative analyses by Le Roy demonstrated superior donor microbiota engraftment in 3-week-old weaned SPF micecompared to 8-week-old adults (Le Roy et al., 2018). This may be because animals with low gut microbiota richness exhibit superior engraftment efficacy (Ericsson et al., 2017), as microbial diversity naturally increases with age (Zhang et al., 2015). By contrast, the dietary transition to solid food during weaning generates transient microbial instability (Zhang et al., 2015) that requires 11–15 days to achieve full community stabilization (Schloss et al., 2012). Other compelling evidence has demonstrated that microbiota alterations established during juvenile stages are sustained into adulthood and induce phenotypic convergence between host organisms and donor profiles (Cox et al., 2014). Collectively, these findings suggest that 3-week-old or weaning-stage juvenile animals may represent the optimal candidates for FMT selection. However, given the critical role of microbiota-immune crosstalk in host immunological maturation (Al Nabhani et al., 2019), studies advocate using 6–8-week-old adult animals with fully developed immune systems (Crouzet et al., 2013; von Klitzing et al., 2017b; Wrzosek et al., 2018; Daharsh et al., 2019; Xia et al., 2019; Aluthge et al., 2020; Basson et al., 2020; Zhang et al., 2020; Huang et al., 2020; Lin et al., 2021; Mao et al., 2021; Han et al., 2021; Sun et al., 2022; Salandre et al., 2023; Spatz et al., 2023; Liu et al., 2023; Togao et al., 2023; Fan et al., 2023). Although this age-specific model better recapitulates microbiota-mature immune system interactions, it may compromise the efficiency of colonization. Therefore, in HMA model, we recommend strategic selection based on research priorities: juvenile models for microbiota colonization dynamics, and adult animals when investigating immunomodulatory mechanisms.

**Table 4:** Fecal microbiota transplantation (FMT) regimens and colonization efficacy.

Recipient	Age	Gender	Recipient preparation	FMT regimen			Observation time and colonization efficacy	References
				Method	Dose	Duration		
Fischer 344 Rat	Adult	Male	Germ-free(GF)	Oral-gastric gavage	1 mL	Single-dose		(Le Bihan et al., 2015)
Fischer 344 Rat	8w	Male	GF	Oral-gastric gavage	2 mL (10^9^ CFU/mL)	Single-dose		(Crouzet et al., 2013)
Wistar Rat	7w	Male	Antibiotic-induced intestinal microbiota depletion	Oral-gastric gavage	2 mL	Once daily for 21 consecutive days		(Zhan et al., 2024)
Sprague Dawley (SD) Rat	8w		GF	Oral-gastric gavage		Every 2 days for 3 times		(Mao et al., 2021)
SD Rat	10w	Male	GF	Oral-gastric gavage	1 mL (2.7–5.5 × 10^9^ cells)	Single-dose	PCR-coupled denaturing gradient gel electrophoresis: 55.8–64.5% during 2-12w.	(Hanske et al., 2009)
SD Rat	13w	Male	Antibiotic-induced intestinal microbiota depletion	Oral-gastric gavage	0.3 mL (100mg/ml)	Once daily for 3 consecutive days, and twice weekly during the subsequent study period	16S rRNA gene sequencing: at the end of the study (59 days after FMT), 40% of the taxa from human donors engrafted into recipient rats.	(Grabrucker et al., 2023)
A/J strain Mouse	7 w	Male	Antibiotic-induced intestinal microbiota depletion	Oral-gastric gavage		Once a week for12 weeks	16S rRNA gene sequencing:76% and 66% of the mouse sequence mass was reflected in the corresponding human donor sample after 12w.	(Hintze et al., 2014)
C57BL/6JMouse	3-4w	Male	GF	Oral-gastric gavage	0.5 mL	Single-dose		(Chiu et al., 2017)
C57BL/6 Mouse	5w		GF	Oral-gastric gavage	0.1 mL (100 mg/mL)	First dose, 1-week interval repeat	16S rRNA gene sequencing: after 8 weeks, the amplicon sequence variant (ASV) colonization efficiencies were 52%, 52%, 49%, and 49%. The colonization efficiencies at the genus level were 58%, 68%, 66%, and 66%.	(Hutchison et al., 2024)
C57BL/6 Mouse	5-6w	Female and Male	GF	Oral-gastric gavage	0.1 mL	First dose, 2-week interval repeat		(Hsu et al., 2023)
C57BL/6 Mouse	6w	Male	Antibiotic-induced intestinal microbiota depletion	Oral-gastric gavage	0.2 mL	Once daily for 14 consecutive days	16S rRNA gene sequencing:4 genera (i.e., *Oscillospira*, *Enterobacteriaceae*, *Bacteroides*, and *Bacteroidaceae*) enriched in donor feces were successfully transplanted to recipient mice after 2w.	(Xia et al., 2019)
C57BL/6J Mouse	6w	Female	Antibiotic-induced intestinal microbiota depletion	Oral-gastric gavage	0.3 mL	Once every other day, for 3 weeks	16S rDNA Amplicon Pyrosequencing: at 3 weeks post-transplantation, the Bacteroidetes/Firmicutes ratio (B/F ratio) was measured as an indicator of gut microbiota composition. The values for the control group, HMA mice group, and human donor feces were 0.968, 0.482, and 0.267, respectively, indicating that the gut microbiota of transplanted mice closely resembled that of the human donor samples.	(Sun et al., 2022)
C57BL/6J Mouse	6w	Female	Polyethylene glycol-mediated gut decontamination	Oral-gastric gavage	0.35 mL	Once a week for 3 weeks		(Spatz et al., 2023)
C57BL/6 Mouse	6-8w	Female	Antibiotic-induced intestinal microbiota depletion	Oral-gastric gavage	0.2 mL	Once daily for 3 consecutive days		(Salandre et al., 2023)
C57BL/6 Mouse	8w	Male	Antibiotic-induced intestinal microbiota depletion	Oral-gastric gavage	0.2 mL	Once daily for 3 consecutive days in the first week, and every other day to reinforce colonization for the remaining 7 weeks.		(Liu et al., 2023)
C57BL/6J Mouse	8w	Female	Polyethylene glycol-mediated gut decontamination	Oral-gastric gavage	0.2 mL	Twice weekly for 4 weeks	16S rDNA Amplicon Pyrosequencing: human bacteria are detected in recipient mice four weeks after FMT.	(Wrzosek et al., 2018)
C57BL/6J Mouse	8w	Female	Antibiotic-induced intestinal microbiota depletion	Oral-gastric gavage	0.3 mL	Once daily for 2 consecutive days		(von Klitzing et al., 2017a)
C57BL/6J Mouse	8w		GF	Oral-gastric gavage	0.2 mL	Twice daily for 14 consecutive days		(Huang et al., 2020)
C57BL/6 Mouse	6 m		GF	Oral-gastric gavage	0.2 mL (10^9^ CFU/ml)	Single-dose	16S rRNA gene sequencing: 66% (76/115) and 65% (75/115) of healthy donor genus-level taxa were detected in the recipient mice at weeks 1 and 5, respectively.	(Ye et al., 2023)
C57BL/6 Mouse			GF	Oral-gastric gavage	0.2 mL	Single-dose		(Tintelnot et al., 2023)
C57BL/6J Mouse		Male	Antibiotic-induced intestinal microbiota depletion	Oral-gastric gavage	0.2 mL	3 times per week for 21 consecutive days		(Zhang et al., 2023)
C57BL/6N Mouse		Female	GF	Oral-gastric gavage	0.2 mL	Single-dose/Once daily for 4 consecutive days/Once weekly for 4 weeks		(Van Den Ham et al., 2023)
BALB/c Mouse	4w	Female and Male	GF	Oral-gastric gavage	0.5 ml	Single-dose		(Kibe et al., 2005)
BALB/c Mouse	6w	Male	GF	Oral-gastric gavage	0.2 mL	Twice a week	16S rRNA gene sequencing: 70% of genera detected in the human fecal samples were also found in the recipient mice.	(Lin et al., 2021)
BALB/c Mouse	6w	Male	GF	Oral-gastric gavage	0.2 mL	Single-dose		(Togao et al., 2023)
BALB/c Mouse	8-10w		GF	Oral-gastric gavage	0.1 mL	three times a day, at least 4 days		(Zabolotneva et al., 2023)
C3H/HeN Mouse	3w	Female and Male	GF	Oral-gastric gavage	0.2 mL	First dose, 1-week interval repeat	16S rRNA gene sequencing: only 9 (33%), 15 (55%), and 10 (37%) of the 27 shared core ASVs from all donors colonized in the HMA mice.	(Aluthge et al., 2020)
C3H/HeN Mouse	13w	Female	GF		0.1 mL (10^7^ bacteria)	Single-dose	16S rRNA gene sequencing: fecal samples remained recoverable after 12-month cryostorage and successfully colonized the gastrointestinal tract of germ-free recipient mice.	(Reygner et al., 2020)
CD1 Mouse	18w	Female	Antibiotic-induced intestinal microbiota depletion	Oral-gastric gavage	0.2 mL	Once daily for 3 consecutive days		(Sánchez-Quintero et al., 2023, 2022)
db/db Mouse	8w	Male		Oral-gastric gavage	0.2 mL	Once daily for 14 consecutive days	Fluorescence microscopy of Detection: The fecal bacteria solution was stained with the fluorescent dye, and fluorescent signals in the fecal bacteria solution of mice on day 14 confirmed successful colonization.	(Han et al., 2021; Zhang et al., 2020)
KM Mouse	3-4w	Male	GF	Oral-gastric gavage	0.3 mL	Single-dose	16S rRNA gene sequencing: Evaluated by OTUs overlap between HMA mice and human donor (reference normalized to 100%):67.50, 69.61, and 70.00% for the coarse-feed diet-fed mice and 74.42, 85.96, and 72.69% for the purified feed diet-fed mice at 1, 2, and 4 weeks, respectively.	(Dong et al., 2021)
NSG Mouse	6–8 w		Antibiotic-induced intestinal microbiota depletion	Oral-gastric gavage	0.2 mL	First dose,24-hour interval repeat		(Daharsh et al., 2019)
SAMP Mouse	7w		GF	Oral-gastric gavage	0.20 mL/10 g (10^8–9^ CFU/mouse)	Once weekly for 60 days		(Basson et al., 2020)
Swiss-Webster Mouse	5-9w		GF	Oral-gastric gavage	0.1 mL	Single-dose	16S rRNA gene sequencing: 59% to 81% of human-associated bacterial phylotypes (OTUs) were successfully transplanted in mice.	(L. Liu et al., 2022)
Swiss-Webster Mouse	6W	Female	GF	Oral-gastric gavage	0.2 mL	First dose, 3 days after the second gavage	16S rRNA gene sequencing:45 donor-ASVs (53%) were successfully engrafted in recipients.	(Fan et al., 2023)
Swiss-Webster Mouse	8-15w	Female	GF	Oral-gastric gavage	0.2 mL	Single-dose		(Wahlström et al., 2017)
Germ-free gnotobiotic (Gn) pigs	2w		GF	Oral-gastric gavage	1 mL Fecal inoculation blended with 40 mL sterile infant milk formula	Single-dose	16S metagenomic: a similar microbiota composition (>99%) was observed in HMA pigs, at the genus level.	(Renu et al., 2022)
Piglets	6w	Female and Male	GF	Oral via feed bowl admixture		First dose, 14 days after the second gavage	16S rRNA gene sequencing: 24 (89%), 25 (93%) and 19 (70%) of the 27 shared core ASVs from all donors colonized in the HMA piglets.	(Aluthge et al., 2020)
Piglets	2w	Female and Male	GF	Oral	5mL Fecal inoculation blended with 40 mL sterile infant milk formula	Once weekly for 3 consecutive weeks		(Dhakal et al., 2019)
Piglets	5d/8d/23d/30d			Oral-gastric gavage	First 10 mL of 0.2 M carbonate buffer pH 9.5 orally, followed by 3 mL of stool homogenate.	Single-dose		(Zhang et al., 2013)
Altered Schaedler Flora (ASF) C57BL/6 Mouse		Male	ASF	Oral-gastric gavage	0.1 mL (10^10^ cells)	Single-dose		(Staley et al., 2017)

### 3.3 Dietary impact

Dietary modulation plays a pivotal role in shaping the gut microbiome (Zmora et al., 2019). Empirical evidence has confirmed that different diets influence both the composition and function of intestinal microorganisms in humans as well as animals (Beam et al., 2021). This principle is equally applicable to HMA animals. Turnbaugh et al. proved that high-fat high-sugar diets rapidly remodeled the microbiota architectures of HMAs, impaired donor microbiota engraftment, and induced phenotypes associated with metabolic obesity (Turnbaugh et al., 2009). Dietary heterogeneity constitutes a critical determinant that prevents HMA animals from fully replicating the gut microbial profiles of their donors (Silley, 2009). Comparative studies have revealed that donor-matched diets fail to enhance gut microbial engraftment efficiency in HMA mice vs fixed-formula grain-based chows (Van Den Ham et al., 2023). By contrast, Schoeler et al. demonstrated superior microbiota transfer success rates in HMA mice that received analog diets identical to those of their human donors (Schoeler et al., 2024). In a 28-day dietary intervention study, Dong et al. observed equivalent microbial colonization rates between coarse-feed diet (CFD) and purified-feed diet groups (70.00% vs. 72.69%) in HMA mice (Dong et al., 2021). In particular, the CFD-fed mice exhibited gut microbial diversity profiles and functional signatures that demonstrated close proximities to those of their human donors (Dong et al., 2021). Although the effects of standardized feeds on HMA animals remain unclear, current evidence demonstrates that donor-aligned dietary formulations may reduce enteric microbiota discrepancies between donor and recipient ecosystems.

## 4 Experimental administration protocols and treatment duration

Common methods for administering fecal microbiota transplantation (FMT) include rectal enema, co-housing, and oral-gastric gavage. Rectal administration requires anesthetizing the animals, gently inserting a tube into the colon, and slowly injecting a fecal bacteria suspension (Zhou et al., 2019). Nevertheless, this procedure presents technical challenges such as mucosal damage, infection, and uncontrollable absorption efficacy (Bokoliya et al., 2021). Co-housing protocols, which let germ-free (GF) mice be co-housed with colonized mice, are effective for microbiota transfer between conspecifics (Hansen et al., 2012; Seedorf et al., 2014). However, it is not suitable for the establishment of human microbiota-associated (HMA) models (Bokoliya et al., 2021). Oral gastric gavage is a method that involves using a stainless-steel or flexible cannula to a syringe to deliver the fecal suspension directly into the stomach (Bokoliya et al., 2021). This method carries potential complications, including respiratory tract injury, gastric rupture, and weight loss (Bokoliya et al., 2021). Nonetheless, empirical evidence has confirmed that single-dose FMT delivery via gavage reliably induces human microbial colonization in experimental animals (Hanske et al., 2009; Reygner et al., 2020). This approach therefore remains the preferred methodology for establishing HMA models.

Notably, emerging nanotechnology applications have introduced single-cell nanocapsules as a novel delivery vehicle for FMT (Hou et al., 2025). This innovative approach utilizes silk fibroin and phosphatidylcholine to form reinforced nanoshells around intestinal microbiota within 1 hour, achieving microbial encapsulation without compromising viability. Experimental trials involving oral administration of these nanocapsules to GF mice and colitis murine models demonstrated superior performance compared to conventional FMT through three key advantages: (1) protecting microbial communities from gastric acid and pepsin degradation; (2) significantly enhancing microbial engraftment efficiency; and (3) providing additional anti-inflammatory benefits while preserving intestinal epithelial integrity (Hou et al., 2025).

Another critical aspect that merits attention is the dosage and frequency of fecal suspension administration (**Table 1**). The typical standard gavage volumes are 1–2 mL for rats (Hanske et al., 2009; Crouzet et al., 2013; Le Bihan et al., 2015) and 0.1–0.5 mL for mice (Chiu et al., 2017; Han et al., 2021; Liu et al., 2022). For developing pig HMA models, the sparse existing literature on the subject suggests an ideal inoculum volume of 1 mL (Dhakal et al., 2019; Renu et al., 2022; Zhang et al., 2013). Furthermore, some studies have characterized the total number of cells administered within these volumes, reporting, for instance, 1 mL (2.7–5.5 × 10^9^ cells) for SD rats (Hanske et al., 2009), 0.2 mL (10^9^ CFU/ml) for C57BL/6 mice (Ye et al., 2023), 0.1 mL (10^7^ bacteria) for C3H/HeN mice (Reygner et al., 2020), and 0.20 mL/10 g (10^8–9^ CFU/mouse) for SAMP mice (Basson et al., 2020). Administration frequencies vary widely, ranging from single-bolus delivery to daily regimens (1–3 doses/day) spanning 2–60 days, or periodic administration at 2–7-day intervals (Basson et al., 2020; Daharsh et al., 2019; Fan et al., 2023; Wahlström et al., 2017; Zabolotneva et al., 2023; Zhan et al., 2024). Hanke et al. demonstrated that HMA rats exhibited 55.8–64.5% gut microbial similarity to their human donors at 2–12 weeks post-FMT, with no significant differences observed between time points (Hanske et al., 2009). Despite the variations present in murine strains, studies by (Liu et al., 2022) (Ye et al., 2023), and (Dong et al., 2021) demonstrated that fecal suspension doses of 0.1, 0.2, and 0.3 mL achieved colonization efficiencies of 59–81% (operational taxonomic unit, OTU level), 65–66% (genus level), and 67.5–85.96% (OTU level), respectively. The relationship between dosage and engraftment efficiency has yet to be elucidated. Nevertheless, current studies consistently demonstrate ≥50% donor microbiota retention in HMA models following single-dose FMT following adequate intestinal preparation, regardless of the volume administered (Dong et al., 2021; Hanske et al., 2009; Liu et al., 2022; Ye et al., 2023).

Thus, the question has arisen of whether chronic FMT protocols with increased frequency can optimize colonization success has garnered significant attention. Experimental protocols by Aluthge et al. revealed that a secondary 0.2 mL fecal transplant in C3H/HeN mice (delivered at a 2-week interval) induced >96% amplicon sequence variant (ASV)-level microbial retention (Aluthge et al., 2020). By contrast, Hutchison et al.’s cohort of C57BL/6 mice, who received multiple 0.1 mL doses at 7-day intervals, exhibited 49–52% ASV and 58–68% genus-level colonization fidelity (Hutchison et al., 2024). The twice-weekly administration of a 0.2 mL fecal suspension to BALB/c mice revealed 70% genus-level colonization efficiency via 16S rRNA gene sequencing (Lin et al., 2021). Although FMT protocols with increased administration frequency appear to improve colonization success, experimental outcomes varied substantially across the above study. A comprehensive study by Van Den Ham et al. evaluated three fecal transplant schedules (single-dose, 4-day consecutively, and once a week for 4 weeks) using 0.2 mL inocula administered to GF mice (Van Den Ham et al., 2023). The once a week for 4 weeks protocol demonstrated superior colonization efficiency vs the other interventions, which was attributed to its capacity to establish a stabilized intestinal condition that minimized pre-engraftment microbial fluctuations, thereby narrowing the donor-recipient microbiota divergence (Van Den Ham et al., 2023). Another comparative study evaluated four FMT strategies in Polyethylene glycol (PEG)-cleansed C57BL/6J mice: (1) a single round during the first week; (2) two rounds of FMT in the first week; (3) once a week for four weeks; and (4) twice a week for four weeks (Wrzosek et al., 2018). After four weeks, the results showed that: (1) a single FMT enabled the observation of human-derived microorganisms; (2) two rounds of FMT in the first week allowed for the engraftment of sub-dominant human bacteria; (3) once-weekly regimen for four weeks was sufficient to establish dominant bacterial populations; (4) in contrast, FMT twice weekly for four weeks disrupted the stability of the newly established microbial ecosystem (Wrzosek et al., 2018). Therefore, the above evidence supports administering multiple FMT doses (cumulatively ≥2 doses) over a 2–4weeks period during the HMA model preparation to optimize the efficacy of donor microbiota engraftment.

## 5 Microbial colonization assessment strategies

The assessment of donor microbiota engraftment efficiency is performed through diverse detection modalities. These modalities include conventional culturing (Hirayama et al., 1995), next-generation sequencing (NGS) (Kong et al., 2023; Wensel et al., 2022), selective culturing (Chiu et al., 2017), fluorescence *in situ* hybridization (Gérard et al., 2004), and temporal-temperature gradient gel electrophoresis (Respondek et al., 2013). Cultivation and isolation represent conventional approaches wherein microbiota are taxonomically enumerated post-development on selective media. Intrinsic limitations persist as slow-growing or fastidious bacteria, which are subject to microbial competition and stringent nutrient requirements, often resist *in vitro* isolation and cultivation (Kato et al., 2018). Strategies such as oligotrophic media, extended incubation periods, and anaerobic culturing conditions have been implemented to address these limitations (Goodman et al., 2011; Pulschen et al., 2017). Nevertheless, their high level of technical demand exacerbates in the challenges inherent to culturing gut microbiota. The advent of culture-independent NGS has resolved these obstacles by facilitating the direct sequencing of microbial DNA and RNA, thereby facilitating the detection of unculturable bacterial taxa. The emergence of culture-independent NGS has addressed these challenges by enabling the amplification and direct sequencing of microbial DNA and RNA, which in turn enhances the identification of unculturable bacterial taxa (Wensel et al., 2022). Microbial colonization efficacy can be quantified using three principal approaches derived from sequencing data. The first is donor-specific retention percentages calculated using operational taxonomic unit (OTU) (Knights et al., 2011) or amplicon sequence variant (ASV) (Gray et al., 2024) classification systems. Studies have revealed that OTU-based calculations systematically overestimate colonization efficiency vs ASV-resolution analyses (Gray et al., 2024). This might be because OTU analysis provides more spurious taxa (Reitmeier et al., 2021). Consequently, the assessment of colonization efficiency at this tier remains contentious, warranting genus-level analysis (Ye et al., 2023) or the implementation of alternative assessment methodologies. The second approach comprises monitoring the emergence of donor-enriched or species-specific bacterial taxa in recipient microbiota (Xia et al., 2019). This approach faces validity challenges related to interspecies microbial overlap (e.g., *Prevotella*, *Bacteroides*, *Clostridium*, and *Eubacterium*-dominant genera across human, murine, and porcine gut communities) (Li et al., 2018), rendering FMT-dependent colonization indistinguishable from native microbiota. The third approach involves assessing microbial transfer via abundance ratios (e.g., the *Bacteroidetes*/*Firmicutes* ratio) (Sun et al., 2022). However, such evaluations lack diagnostic precision, owing to multifactorial influences on microbial abundance and pre-existing microbial overlap between donors and recipients.

## 6 Conclusion and prospects

**Figure 2:**
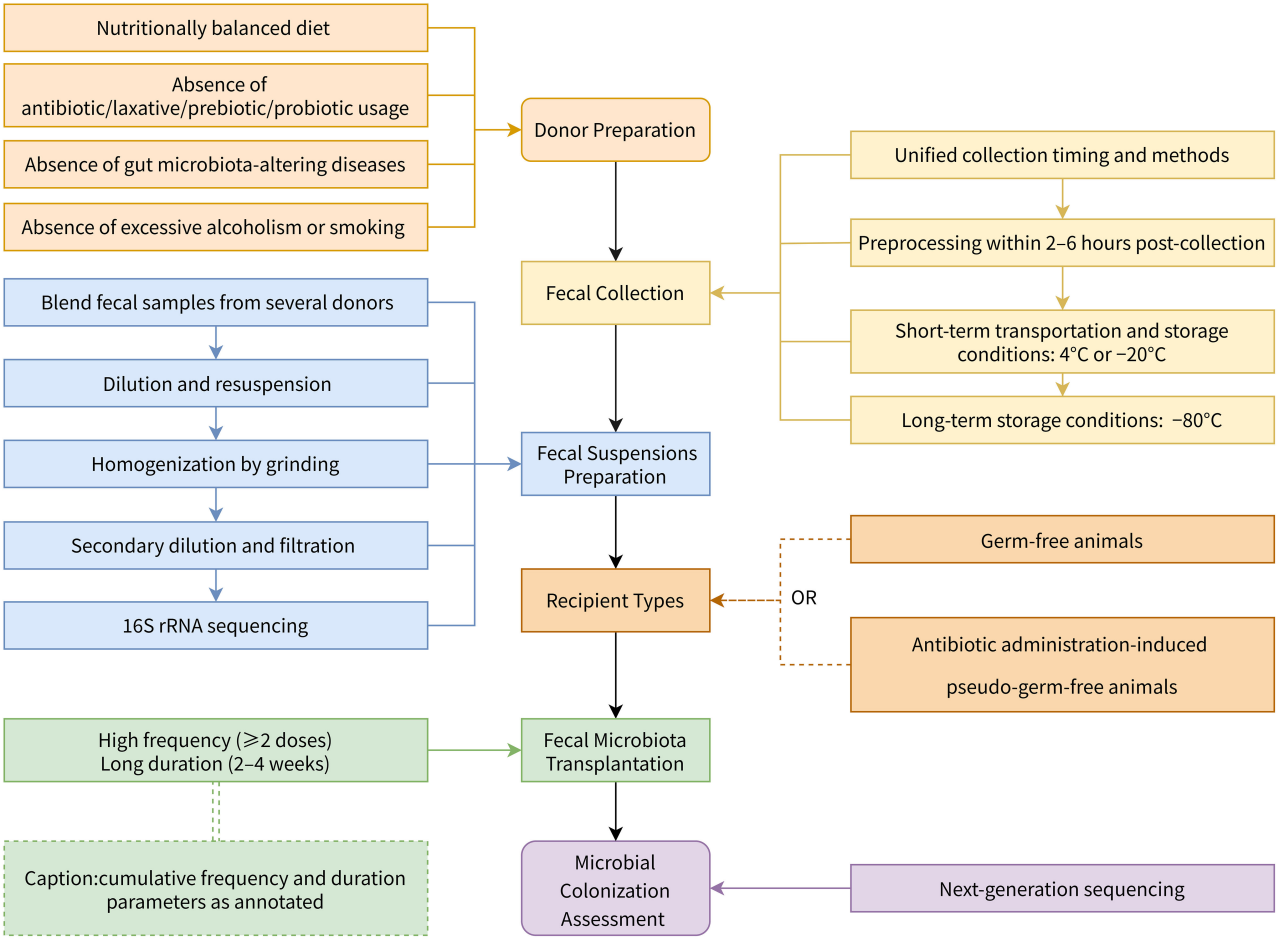
Optimal protocol for establishing human microbiota-associated (HMA) animal models.

HMA animal models serve as indispensable tools for deciphering the roles of microbes in states of both health and disease, by simulating humanized gut microbiomes. The core technical aspects underlying the construction of HMA models remain under investigation. This review of the critical elements involved in the development of HMA models has delineated the following key findings(**Figure 2**): (1) Donor screening necessitates rigorous interviews regarding dietary habits, medication history, and pre-existing pathologies to eliminate a host of factors that can influence gut microbial composition. (2) Fecal preservation mandates immediate refrigeration within a 2–6 h window after collection. (3) Fecal suspension preparation should employ multi-donor blending strategies coupled with 16S rRNA sequencing to verify microbial composition. (4) Recipient selection should preferentially utilize adult germ-free (GF) or antibiotic-induced pseudo-GF animals that are fed diets matching their human donors. (5) Oral gavage represents the ideal route for FMT, with protocols utilizing high administration frequencies (cumulatively ≥2 doses) and extended durations (2–4 weeks) demonstrating significantly higher engraftment rates. (6) Next-generation sequencing (NGS) represents an efficient methodology for quantifying microbial engraftment. Metrics used include retention rates of operational taxonomic unit (OTU)/amplicon sequence variant (ASV) between donor and recipient microbiomes. Other metrics involve the detection of donor-specific bacterial strains, and phylum-level abundance ratios. These findings establish a methodological foundation for standardizing HMA model generation protocols. The development of HMA models faces persistent challenges that include objective microbial disparities between donors, unstable colonization of human microbiota in animal recipients, methodological variations in recipient animal preparations, and various dietary influences on microbial colonization. Moreover, a rational method for assessing the efficiency of colonization is needed to maximize the preparation of reproducible and representative HMA models.
